# Supplementary material for: Impact of Providing Peer Support on Medical Students’ Empathy, Self-Efficacy, and Mental Health Stigma
Source: Int J Environ Res Public Health. 2022 Apr 23;19(9):5135. doi: 10.3390/ijerph19095135 (PMC9099875; doi:10.3390/ijerph19095135)
Supplement: Supplementary file 1 [file ijerph-19-05135-s001.zip › ijerph-1635169-supplementary.pdf]

Supplemental Table S1. Post-Study Survey Responders vs. Non-responders at Baseline.

		Post-Study Survey Responder Status		
		**Yes**	**No**	
		**# (%)**	**# (%)**	***p*-Value**
Total		17 (47.2)	19 (52.8)	
Age (years)		25.12 ± 2.12	25.11 ± 2.56	0.988
Gender				0.505
	Male	8 (47.1)	8 (47.1)	
	Female	8 (47.1)	11 (57.9)	
	Non-binary/Gender Queer	1 (5.9)	0 (0)	
Race				0.156
	Asian	7 (41.2)	3 (15.8)	
	White	7 (41.2)	13 (68.4)	
	Black	0 (0)	1 (5.3)	
	Latino/Hispanic	1 (5.9)	2 (10.5)	
	Other	2 (11.8)	0 (0)	
Med School Year				0.842
	2nd Year	9 (52.9)	11 (57.9)	
	3rd Year	5 (29.4)	4 (21.1)	
	4th Year	3 (17.6)	4 (21.1)	
New Member				0.721
	Yes	11 (64.7)	14 (73.7)	
	No	6 (35.3)	5 (26.3)	
Empathy Score (%)		47.40 ± 12.21	52.29 ± 16.62	0.328
Self-Efficacy Score (%)		70.92 ± 17.70	73.10 ± 16.79	0.712
Stigma Score (%)		33.58 ± 8.65	37.04 ± 11.78	0.328

Welch's t-tests were conducted for continuous variables, and chi-square tests were conducted for categorical variables; significance level of 0.05
